# Enhanced tuberculosis clearance through the combination treatment with recombinant adenovirus-mediated granulysin delivery

**DOI:** 10.7150/thno.48052

**Published:** 2020-08-08

**Authors:** Ling Hao, Jilei Ma, Chunwei Shi, Xiaosong Lin, Yandi Zhang, Banga Ndzouboukou Jo-Lewis, Qing Lei, Nadeem Ullah, Zhongjie Yao, Xionglin Fan

**Affiliations:** 1 Department of Pathogen Biology, School of Basic Medicine, Tongji Medical College, Huazhong University of Science and Technology, Wuhan 430030, People's Republic of China.; 2 Department of Clinical Laboratory, The First Affiliated Hospital of Zhengzhou University, Zhengzhou 450052, People's Republic of China.

**Keywords:** granulysin, tuberculosis, multidrug-resistant, chemotherapy, therapeutic vaccine

## Abstract

**Rationale:** Tuberculosis (TB) remains the leading cause of death among infectious diseases worldwide. Poor compliance of TB patients to the lengthy treatment increases the risk of relapse and leads to the emergence of multidrug-resistant and extensively drug-resistant TB (MDR-TB and XDR-TB). More effective therapies for TB are urgently needed. We hypothesized that granulysin-mediated clearance of *M. tuberculosis* parasited inside and outside of alveolar macrophages in presumptive infected hosts might enhance the chemotherapeutic efficacy on TB.

**Methods:** Recombinant adenovirus type 5 (rAd5) based therapeutic vaccines rAdhGLi and rAdhGLs (rAds) were respectively developed to express intracellular and extracellular granulysin. The *ex vivo* bactericidal effects of rAdhGLi and rAdhGLs were evaluated on U937 and RAW264.7 cells. The efficacy of immunotherapy with both rAdhGLi and rAdhGLs on TB SCID mice, or immunotherapy combined with chemotherapy on drug-susceptible TB or MDR-TB mouse models were further evaluated.

**Results:** rAdhGLs, as well as rAdhGLi, showed a direct bactericidal effect on extracellular or intracellular* M. tuberculosis* H37Rv and MDR-TB clinical strains, respectively. Immunotherapy with a dose of 10^9^ PFU of rAdhGLi and 10^9^ PFU of rAdhGLs demonstrated a more significant bactericidal effect on *M. tuberculosis* H37Rv infected SCID mice and prolonged their survival periods than rAdhGLi or rAdhGLs alone. More importantly, chemotherapy combined with rAds immunotherapy shortened the chemotherapeutic duration to 4 months on *M. tuberculosis* H37Rv infected mice and prevented the relapse. Combination of rAds with chemotherapy on MDR-TB mice also more significantly decreased organ bacterial load than their single use.

**Conclusions:** Delivery of granulysin by recombinant adenovirus to the infected lung could enhance the clearance of TB *in vivo* and might be a promising adjunct therapeutic vaccine for TB and MDR-TB.

## Introduction

Tuberculosis (TB) remains the most threatening infectious disease, resulting in approximately ten million new cases and 2 million deaths worldwide annually 1. The current treatment strategy for drug-susceptible TB cases contains a 6-month regimen of several first-line anti-TB drugs such as rifampin (RFP), isoniazid (INH), and pyrazinamide (PZA) with 2-month initial phase, and following RFP and INH with 4-month continuation phase. However, poor compliance of TB patients to the lengthy treatment leads to the emergence of drug-resistance, as evidenced by that the average rate of resistance to these drugs in previously treated cases was high as 43% 1. Moreover, multidrug-resistant and extensively drug-resistant *M. tuberculosis* (MDR-TB and XDR-TB) strains have been isolated from 5.58% of new diagnosed TB patients annually 1. Longer, more expensive and more complex treatments are required for these patients, but the cure rate is below 50% 2, 3. Clearly, more effective therapies for TB are urgently needed.

Granulysin is expressed by activated natural killer cells, γδ T cells, and cytotoxic T lymphocytes 4, and colocalizes in cytolytic granules such as perforin and granzymes with a wide range of antimicrobial activities 5. Granulysin was confirmed to be capable of killing drug-susceptible as well as drug-resistant strains by altering the membrane integrity of *M. tuberculosis in vitro* 5-8. Previously, a recombinant replication-deficiency adenovirus type 5 (rAd5) based therapeutic vaccine rAdhGLi was developed to express intracellular granulysin, which demonstrated a direct killing effect on *M. tuberculosis* parasited in the macrophage *ex vivo* and also showed a significant therapeutic effect on TB mouse models via inhalation route 9. We hypothesized that granulysin-mediated clearance of* M. tuberculosis* parasited inside and outside of alveolar macrophages in presumptive infected hosts might enhance the chemotherapeutic efficacy on TB. To this end, rAdhGLs, the granulysin delivered as a secretory form by rAd5 was firstly constructed in this study, and then the adjunctive chemotherapy effects of both rAdhGLi and rAdhGLs on drug-susceptible TB and MDR-TB were evaluated in murine models.

## Materials and Methods

### Ethical Statement

All animal experiments were performed in accordance with the guidelines of the Chinese Council on Animal Care. The research protocols were approved by the Committee on the Ethics of Animal Experiments of Tongji Medical College, Huazhong University of Science and Technology.

### Preparation of recombinant adenovirus vectors expressing granulysin

The recombinant shuttle plasmid pDChGLs, containing the tandem-fusion of the sequence encoding the signal peptide of tissue plasminogen activator (tPA) and human granulysin (238 bp) was synthesized by Obio Technology Company (Shanghai, China). As shown in **Figure S1A**, rAd5-based rAdhGLs, expressing granulysin extracellularly, was packaged and constructed by co-transfecting plasmids pDChGLs and pBHGloxΔE1,3Cre (Microbix, Mississauga, ON, Canada) (1:1) into human embryonic kidney 293 (HEK293, ATCC^®^ CRL-1573 ^TM^) cells with Lipofactamine 2000 reagent (Invitrogen, Carlsbad, CA, USA), as previously described 9, 10. rAdhGLs was rescued by homologous recombination, amplified in HEK293 cells, and purified with the standard method of CsCl density gradient centrifugation. The titer for rAdhGLs was detected by an Adenovirus Rapid Titer Testing kit (Neuron Biotech, Shanghai, China) and displayed as PFU/mL.

HEK293 cells were firstly infected with rAdhGLs at a multiplicity of infection (MOI) of 10 (virus:cell) for 48 h. Quantitative real-time PCR (qRT-PCR) technology was used to detect the expression of granulysin mRNA (**Table S1**). The relative expression level of mRNA was normalized to human β-actin and calculated using the 2^-ΔΔCt^ method. Western blotting was further explored to confirm the secretory expression of granulysin. In brief, U937 (human histiocytic lymphoma cell line) or RAW264.7 (mouse leukemic monocyte macrophage cell line) cells, obtained from the Cell Bank of the Chinese Academy of Sciences (Shanghai, China), were treated with rAdhGLs at MOIs of 2000:1 and 300:1 (virus:cells) for 72 h, respectively. Culture supernatants and cell lysates were separately used to verify the expression of granulysin by western blotting with mouse anti-granulysin antibody (Santa Cruz Biotechnology, Dallas, TX, USA; Cat no. sc-271119) as the primary antibody and peroxidase conjugated Goat anti-mouse IgG (H+L) (Proteintech, Chicago, IL, USA; Cat no. SA00001-1) as the secondary antibody. rAdhGLi or wild-type Ad5 AdNull (Obio Tech.) were used as controls.

### The proliferation and death of rAd-treated U937 and RAW264.7 cells

The proliferation and death of rAd-infected U937 or RAW264.7 cells were analyzed as previously described 9. In brief, U937 or RAW264.7 cells were treated with rAd at MOIs of 2000:1 and 300:1 (virus: cells), respectively. After 0, 24, 48, 72 and 96 h, a Cell Counting Kit (Zoman Biotech, Beijing, China) was used to test the cell proliferation. Alternatively, cells were stained using an Annexin V-APC/7-AAD kit (Keygen Biotech, Nanjing, China) to label the apoptotic cells and analyzed by a BD FACSCanto flow cytometer (BD Biosciences, San Jose, CA, USA). Culture medium or AdNull were used as negative controls. The results were analyzed by FlowJo software (Tree Star Inc., Ashland, OR, USA) and displayed as the Mean ± SD of three repeats.

### *Ex vivo* bactericidal effects

10^6^ of U937 or RAW264.7 cells were seeded in each well of 6-well plates in triplicate and infected with ~10^3^ CFU of MDR-TB strain for 24 h, and then treated with rAdhGLi for another 96 h as previously described 9. Culture medium, AdNull and rAdhGLs were used as controls. Alternatively, cells were first treated with rAdhGLs for 72 h. Culture supernatant was then collected with 10-fold serial dilutions, and co-incubated with ~10^4^ CFU of *M. tuberculosis* H37Rv or ~10^3^ CFU of a clinical MDR-TB strain (No. WPH2016) for 24 h, respectively. Culture medium and the secretory products from AdNull or rAdhGLi were used as controls. At the end of treatments, the survival bacteria were enumerated on Middlebrook 7H11 agar plates (Difco Laboratories, Sparks, MD, USA) enrichment with 10% ADC (Difco Laboratories) and cultured at 37 °C for 3 to 4 weeks.

### Therapeutic effects on *M. tuberculosis*-infected SCID mice

Female SCID mice aged 6-8 weeks (HFK Bioscience, Beijing, China) were first infected intranasally (i.n.) with ~200 CFU of virulent *M. tuberculosis* H37Rv strain as previously described 9. Two weeks post-challenge, a dose of 10^9^ PFU rAdhGLi and/ or 10^9^ PFU rAdhGLs in 25 μl PBS was given i.n. to mice once, respectively. PBS and 10^9^ PFU of AdNull were used as negative controls. Two weeks later, ten mice in each group were sacrificed to measure bacterial loads in the lung and spleen and lung pathological changes were compared. To detect the survival rate, twenty mice in each group were under surveillance after infection through the entire observation period and death was recorded daily.

### Therapeutic effects on drug-susceptible TB mice

Specific pathogen free female C57BL/6 mice, aged 6-8 weeks (Center for Animal Experiment of Wuhan University, Wuhan, China) were first infected i.n. with ~100 CFU of *M. tuberculosis* H37Rv strain. 18 days post-infection, six mice were sacrificed to provide baseline values before treatments by detecting bacterial load in both lung and spleen. Infected mice were randomly grouped and received chemotherapy with or without an inhalation of rAdhGLi, rAdhGLs, or the combination (**Table S2**). A dose of 10^9^ PFU rAdhGLi and/ or 10^9^ PFU rAdhGLs in 25 μl PBS was given i.n. to infected mice once at the beginning of the treatment. Mice received chemotherapy with 2RHZ/4RH, containing 2-month initial phase of with 10mg/kg/day of RFP (Sigma), 25mg/kg/day of INH (Sigma), and 150mg/kg/day of PZA (Sigma), followed by 4-month continuation phase with RFP and INH. Drugs were administered 5 days/week by gavage. PBS was used as controls. 2, 4 and 6 months after treatments, ten mice in each group were sacrificed to assess the treatment efficacy, and the other ten mice were kept for three more months without any treatments for culture conversion.

### Therapeutic effects on MDR-TB mice

C57BL/6 mice were first challenged i.n. with ~300 CFU of the MDR-TB clinical strain. 15 days post-infection, six mice were sacrificed as described above to obtain the baseline of organs bacterial load and confirm the establishment of MDR-TB mouse model. Infected mice were randomly grouped and received chemotherapy with or without immunotherapy (**Table S3**). Immunotherapeutic regimens were performed as described above. Mice received the chemotherapeutic regimen CLPPK, including the combination of several drugs such as clofazimine (CFZ, Sigma), levofloxacin (LFX, Sigma), PZA, *p*-aminosalicylic acid (PAS, Sigma), and kanamycin (Kan, Sigma). CFZ (20 mg/kg/day), LFX (200 mg/kg/day), PZA (150 mg/kg/day), and PAS (750 mg/kg/day) were administered 5 days/week by gavage. Kan was administered subcutaneously (s.c.) 100 mg/kg/day for 5 days per week. RHZ or PBS were used as controls. 15, 30, or 60 days after treatments, therapeutic efficacy was compared by the bacterial load per organ and the lung histopathological changes as previously described 9. The pathological scores were obtained by measuring the percentage of the consolidation area of the whole field of vision (magnification ×40), and expressed as the Mean ± SD of five fields of vision from each group.

### Statistical analysis

All data were collected and analyzed by using GraphPad Prism 5.0 (San Diego, CA, USA). A two-group comparison was assessed by a two-tailed student's *t* test. Multigroup analyses were carried out by one-way ANOVA test, and Tukey's multiple comparison test was used for further pair-wise comparison. Survival curves were obtained by the Kaplan-Meier method and compared by log-rank (Mantel-Cox) test. A statistic significant difference was considered as *p*<0.05.

## Results

### rAdhGLs or rAdhGLi alone showed bactericidal effects *ex vivo*

**Figure 1:**
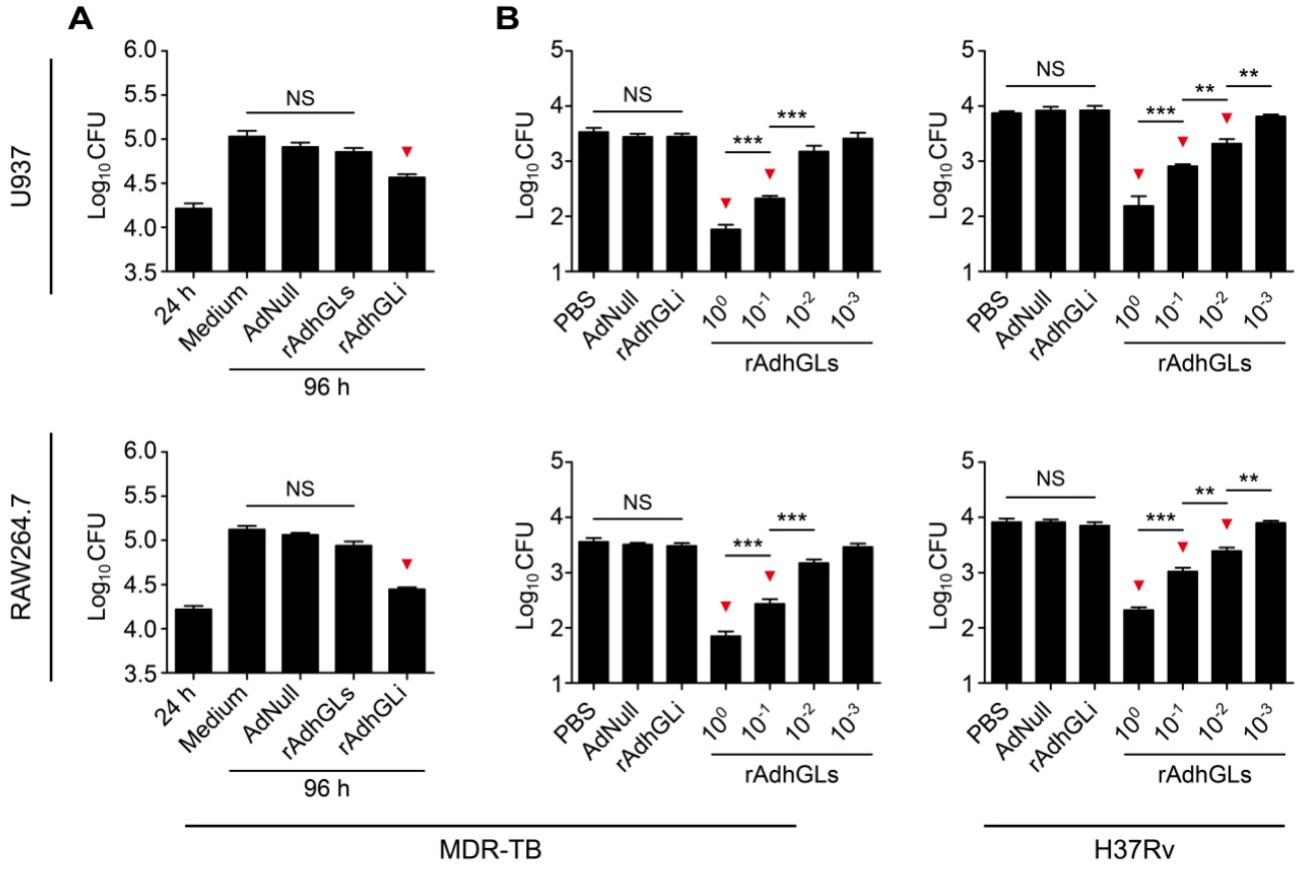
***Ex vivo* killing effect of rAdhGLi and rAdhGLs. (A)** *Ex vivo* killing effect of rAdhGLi on intracellular MDR-TB strain phagocytosed by U937 or RAW264.7 cells. Culture medium, AdNull or rAdhGLs were used as controls. **(B)** Bactericial effect of secretory products from rAdhGLs-treated U937 or RAW264.7 cells on MDR-TB or *M. tuberculosis* H37Rv strains, respectively. Culture medium and the secretory products from AdNull or rAdhGLi were used as controls. The results were shown as the Mean ± SEM (log_10_ CFU) of three repeats. Statistical significance was calculated by One-way ANOVA with Tukey's multiple comparison test. NS indicated not significant, ^**^ indicated *p*<0.01, ^***^ indicated *p*<0.001, and ^▼^ indicated *p*<0.05 when compared with controls.

rAd5-based rAdhGLs was successfully constructed to secret granulysin under the direction of the signal peptide of human tissue plasminogen activator (tPA) (**Figure S1A**). rAdhGLs, as well as rAdhGLi-infected HEK293 cells displayed much higher levels of granulysin mRNA than AdNull did, which indicates that rAdhGLs could express granulysin in infected cells (**Figure S1B**). 72 h post treatment, recombinant granulysin (9 kDa) was only detected in the supernatants of rAdhGLs-treated U937 or RAW264.7 cells by western blotting, which confirms the secretory expression of granulysin (**Figure S1C-D**). Instead, the expression of granulysin was only observed in the lysates of rAdhGLi-treated cells (**Figure S1C-D**), in line with our previous report 9. In particular, rAdhGLs, rAdhGLi, as well as combined infection with rAdhGLs and rAdhGLi, did not impose any obvious effects on proliferation and death of treated U937 and RAW264.7 cells, respectively (**Figure S2**). Previously, rAdhGLi was confirmed to have a direct killing effect on intracellular *M. tuberculosis* H37Rv strain 9. Here, rAdhGLi also more significantly inhibited the growth of the MDR-TB clinical strain in both infected U937 and RAW264.7 cells than culture medium and AdNull controls, respectively (**Figure 1A**). However, rAdhGLs could not inhibit the growth of intracellular *M. tuberculosis*. Interestingly, culture supernatants containing the granulysin, successfully secreted by rAdhGLs-treated U937 or RAW264.7 cells, had a clear dose-dependent growth inhibition on *M. tuberculosis* H37Rv or MDR-TB strains (**Figure 1B**). Together these data confirm that rAdhGLi and rAdhGLs have a bactericidal effect on intracellular, or extracellular drug-susceptible and drug-resistant TB strains, respectively.

### Combination of rAdhGLs and rAdhGLi enhanced the bactericidal effect *in vivo*

**Figure 2:**
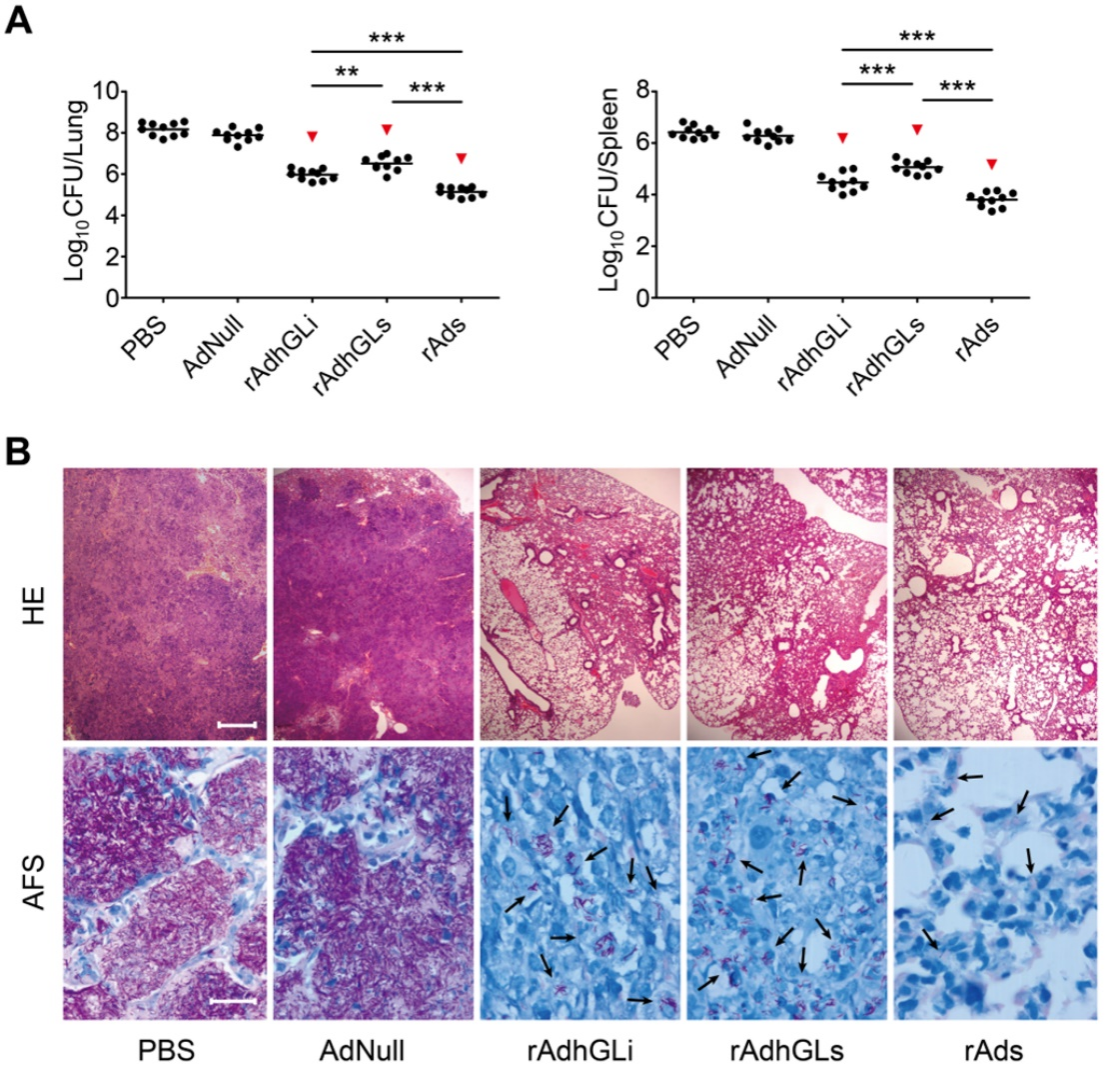
**Therapeutic effect of rAds on SCID mice.** Two weeks after SCID mice were challenged i.n. with ~200 CFU of *M. tuberculosis* H37Rv strain, SCID mice were grouped and given i.n. with a dose 10^9^ PFU of either rAdhGLs, rAdhGLi, or combination once. **(A)** Two weeks later, lung and spleen from each mice were harvested aseptically, and CFU numbers per organ were enumerated and shown as the Mean ± SEM (log_10_ CFU/organ) (n = 10). Statistical significance was calculated by One-way ANOVA with Tukey's multiple comparison test. ^**^ indicated *p*<0.01, ^***^ indicated *p*<0.001, and ^▼^ indicated *p*<0.001 when compared with PBS or AdNull controls. **(B)** Representative lung histopathology visualized by HE (scale bar = 400 µm) and AF staining (scale bar = 20 µm). HE: hematoxylin-eosin; AFS: acid-fast staining. Arrows indicated AF-positive bacteria.

To explore the therapeutic effects of the combination of rAdhGLs and rAdhGLi (rAds), *M. tuberculosis* H37Rv-infected SCID mouse models were established. Two weeks after treatments, the therapeutic efficacy was compared (**Figure 2**). Consistent with previous findings, rAdhGLi alone resulted in a more significant decrease of organs bacterial load than PBS or AdNull controls (**Figure 2A**). Interestingly, rAdhGLs alone also more significantly decreased organs bacterial load than PBS, as well as AdNull controls. However, the bactericidal effect of rAdhGLs alone treated mice was inferior to that of rAdhGLi. More importantly, the combination of rAdhGLs and rAdhGLi provided the strongest therapeutic efficacy of all groups (**Figure 2A-B**).

**Figure 3:**
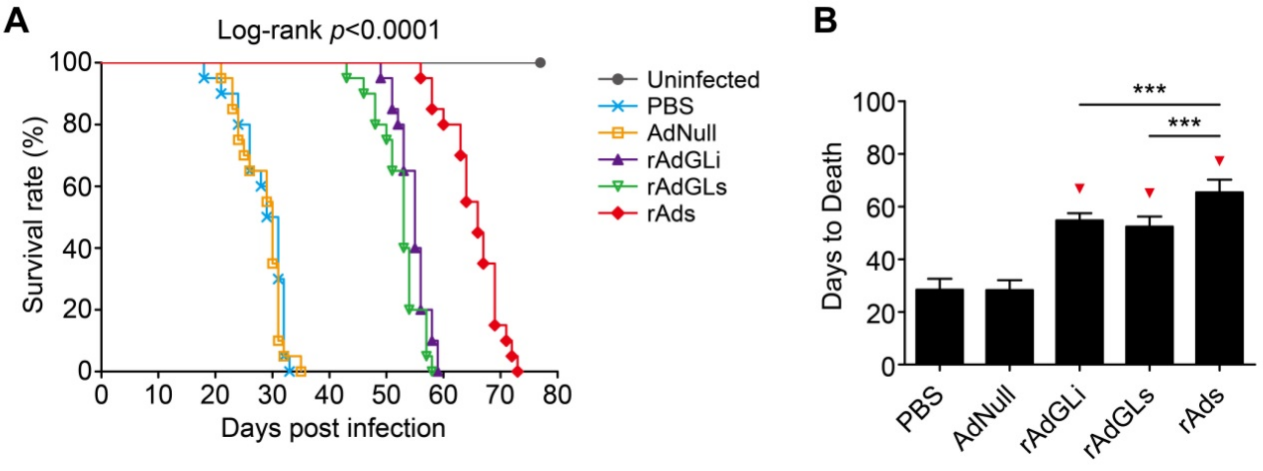
** Combination treatments of rAdhGLi and rAdhGLs improves the survival time of *M. tuberculosis* infected SCID mice. (A)** Animal death events and mortalities were monitored and recorded over the course of 11 weeks post-challenge. Kaplan-Meier survival analysis with log-rank (Mantel-Cox) test was employed to compare the difference of survival rates among 6 groups. **(B)** Comparison of the days to death among different regimens. The results were shown as the Mean ± SD (n = 20). Statistical significance was calculated by One-way ANOVA with Tukey's multiple comparison test. ^***^ indicated *p*<0.001, and ^▼^ indicated *p*<0.001 when compared with PBS or AdNull controls.

The survival of TB-infected SCID mice treated with rAdhGLi or rAdhGLs alone or their mixture was further compared (**Figure 3**). PBS and AdNull treated mice died with similar mean days to death (MDD) of 28.4 ± 0.9 and 28.3 ± 0.8 days. Interestingly, rAdhGLi or rAdhGLs alone more significantly prolonged the survival time of infected SCID mice than PBS and AdNull controls, and their MDDs were 54.8 ± 0.6 and 52.4 ± 0.9 days, respectively. Of all groups, the longest survival time observed in mice received the combination treatment of rAdhGLi and rAdhGLs, with a MDD of 65.4 ± 1.1 days (**Figure 3A-B**). All of these data demonstrate that the combination of rAdhGLs and rAdhGLi enhances bactericidal effects *in vivo* when compared with their single use.

### Combination of rAds shortened the chemotherapeutic duration of drug-susceptible TB

**Figure 4:**
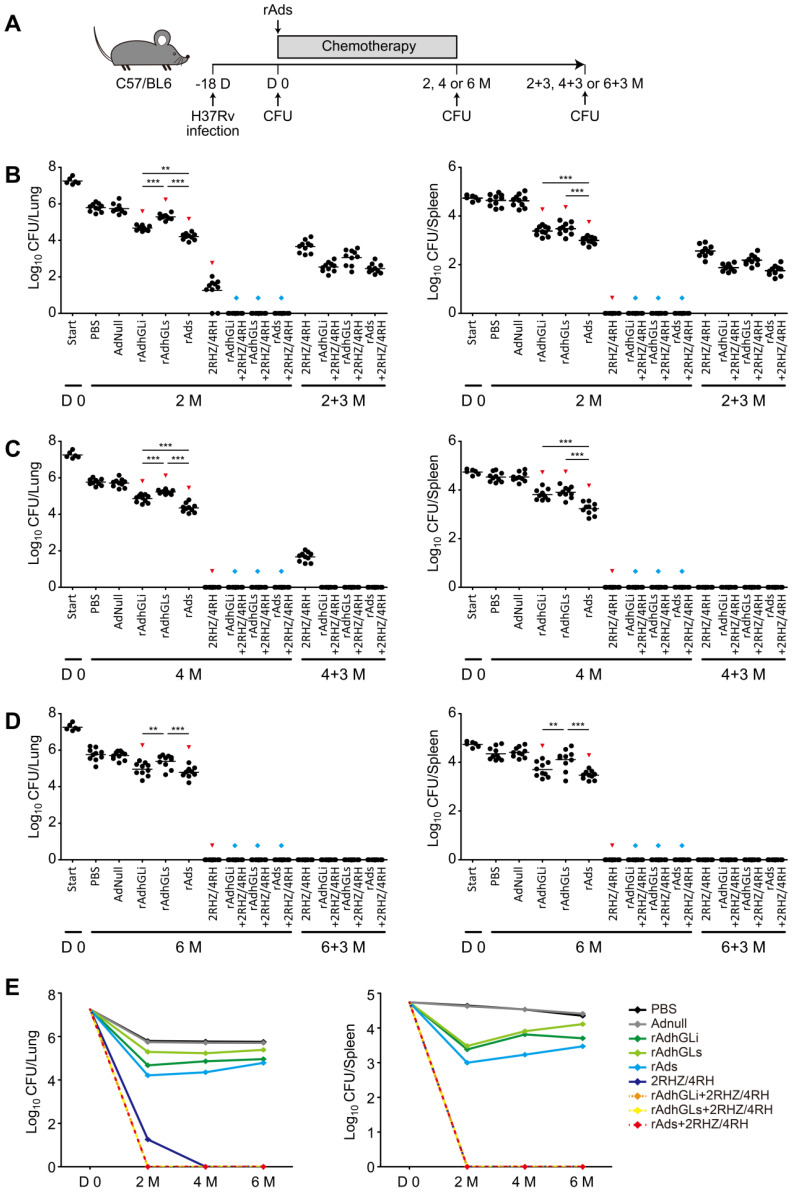
** Treatment efficacy of different regimens on drug-susceptible TB mice. (A)** C57BL/6 mice were infected as previously described. The treatments were done after 18 days post-infection for 2, 4 and 6 months, followed by evaluating the outcomes 3 more months after the end of each treatment. **(B-D)** Bacterial counts from the lungs and spleens of individual mice after receiving 2 **(B)**, 4 **(C)** and 6 **(D)** months treatment were detected. The results were shown as the Mean ± SEM (log_10_ CFU/organ) (n = 10). Statistical significance was calculated by One-way ANOVA with Tukey's multiple comparison test. ^**^ indicated *p*<0.01, ^***^ indicated *p*<0.001, ^▼^ indicated *p*<0.05 when compared with PBS or AdNull controls, and ^◆^ indicated *p*<0.001 when compared with respective rAd single use. 2+3 M, 4+3 M and 6+3 M meant that bacterial load in the lung and spleen was enumerated 3 months after the end of treatments for either 2, 4, or 6 months, respectively. **(E)** Change curves of mean bacterial load per organ in different groups over treatment times.

To explore the therapeutic efficacy of rAdhGLi and rAdhGLs on drug-susceptible TB, C57BL/6 mice were first infected i.n. with ~100 CFU of *M. tuberculosis* H37Rv strain. 18 days post-infection (Day 0), bacterial loads in the lung and spleen reached over 10^7^ and 10^4^ CFU and used as the baseline before treatments, respectively (**Figure 4**). Then, mice were treated with different regimens as described in **Figure 4A**. During the whole experimental period, the highest bacterial loads in both lung and spleen were obtained in PBS or AdNull control mice, of all groups (**Figure 4B-E**). Moreover, rAdhGLi alone or combination of rAds more significantly decreased bacterial loads in both organs than PBS or AdNull control mice at all detected time points. Interestingly, immunotherapy with combination of rAds more significantly inhibited the growth of *M. tuberculosis* in both organs than their single use after 2 or 4 months treatment. However, different immunotherapeutic regimens alone only reduced bacterial load after 2 months treatment, which indicates that these regimens alone could not completely eliminate *M. tuberculosis in vivo* **(Figure 4E)**. When compared the control mice, treatment with 2RHZ resulted in a sharp decrease of bacterial load in the lung and spleen. It was of noteworthy that different immunotherapeutic regimens in combination with 2RHZ did not detect any bacteria in both organs after 2 months of treatment, but culture conversion was occurred in these groups 3 months later (**Figure 4B**). 2RHZ/2RH alone treated mice had culture-negative organs after 4 months of treatment, but relapsed in the lung of all mice 3 months later (**Figure 4C**). In contrast, immunotherapy with different rAds combined with 2RHZ/2RH groups appeared to eliminate *M. tuberculosis* in both organs as earlier as 4-month treatment, and there were negative culture conversions in the lung and spleen of all mice (**Figure 4C**). After six months of treatment, different rAds combined with 2RHZ/4RH groups or 2RHZ/4RH alone had no detectable bacteria in both organs and negative culture conversion (**Figure 4D**). Therefore, our results demonstrated that immunotherapy with the combination of rAdhGLi and rAdhGLs could more quickly shorten the standard chemotherapeutic duration and thwart the relapse.

### Combination of rAds was an effective adjunct for chemotherapy of MDR-TB

**Figure 5:**
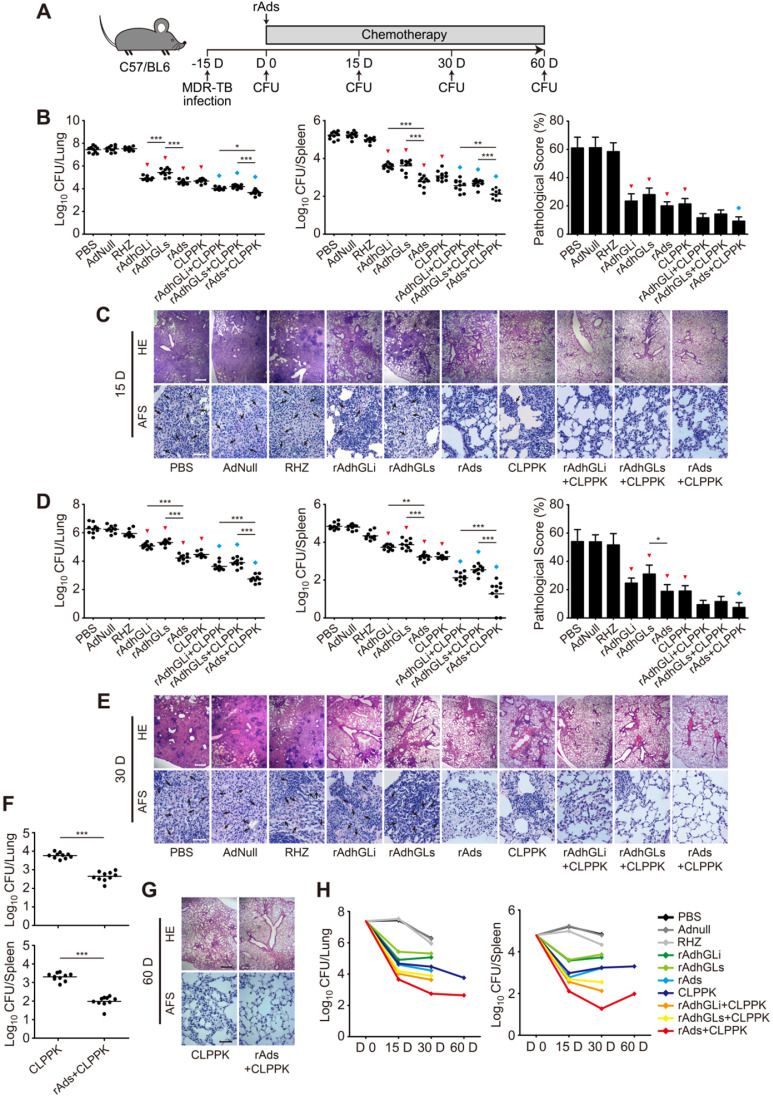
** Adjunctive immunotherapeutic efficacy of rAdhGLi and rAdhGLs with or without chemotherapy on MDR-TB infected mice. (A)** C57BL/6 mice were infected and treated as previously described. The treatments were performed after 15 days post-infection for 15, 30 and 60 days. **(B-G)** After 15 days **(B, C)**, 30 days **(D, E)** and 60 days **(F, G)** of treatments, mice were sacrificed. Bacterial load in the lung and the spleen in different groups were shown as the Mean ± SEM (log_10_ CFU/organ) (n = 10). The representative lung pathological changes of different groups were also visualized by HE (scar bar = 400 µm) and AF staining (scar bar = 50 µm). Pathological scores were shown as the Mean ± SD (n = 3). HE: hematoxylin-eosin; AFS: acid-fast staining. Arrows indicated AF-positive bacteria. Statistical significance was calculated by One-way ANOVA with Tukey's multiple comparison test or by two-tailed unpaired *t* test for only 2 group comparison. ^*^ indicated *p*<0.05, ^**^ indicated *p*<0.01, ^***^ indicated *p*<0.001, ^▼^ indicated *p*<0.001 when compared with PBS or AdNull controls, and ^◆^ indicated *p*<0.05 when compared with respective single use. **(H)** Change curves of mean bacterial load per organ in different groups over treatment times.

To explore the therapeutic effect of rAdhGLi and rdAhGLs on MDR-TB, C57BL/6 mice were first challenged i.n. with ~300 CFU of the MDR-TB strain. MDR-TB mouse models were established successfully as demonstrated by the bacterial load in the lung reached up to 10^7^ CFU 15 days post-challenge. Infected mice were then randomly grouped and given CLPPK with or without immunotherapeutic treatments (**Figure 5A**). After received 15 or 30 days treatment of RHZ alone, AdNull or PBS control, mice had the highest bacterial loads in both lung and spleen of all groups (**Figure 5B, D**). In contrast, CLPPK showed a stronger therapeutic efficacy than the PBS control (**Figure 5B, D**). Immunotherapy with rAdhGLi or rAdhGLs alone also resulted in a more significant decrease of organs bacterial load, when compared to PBS or AdNull controls (**Figure 5B, D**). Moreover, after 30 days of combination treatment with rAds, mice resulted in a lower bacterial load in the lung and spleen than rAdhGLi or rAdhGLs alone did (**Figure 5D**). Interestingly, there was no statistic difference in organs bacterial load between rAds and CLPPK groups after 15 and 30 days of treatments, respectively (**Figure 5B, D**). More importantly, the treatment efficacy of the combination rAdhGLi and/ or rAdhGLs with CLPPK was superior to that of their single use (**Figure 5B-E**). Most importantly, combination rAds with CLPPK had the strongest therapeutic efficiency among all groups, as demonstrated by the lowest bacterial load in both lung and spleen, less lung histopathological changes and much lower scores (**Figure 5B-E**). After 2-month treatments, mice received combination rAds with CLPPK had much lower organs bacterial loads than CLPPK alone (**Figure 5F, G**). In particular, combination rAds with CLPPK showed the strongest ability in the clearance of the MDR-TB strain especially in the early 30 days among all groups (**Figure 5H**). Therefore, immunotherapy with combination of rAdhGLi and rAdhGLs is an effective adjunct for chemotherapy of MDR-TB.

## Discussion

The lengthy 6-month chemotherapy results in TB patients' poor compliance thus attributing to the emergence of MDR-TB and XDR-TB. Therefore, it is very important and urgent to develop new therapeutic regimens to shorten the treatment period or target drug-resistant TB diseases, in order to better control global TB epidemic. Our study demonstrated that delivery of granulysin by recombinant adenovirus to the infected lung via inhalation route could enhance the clearance of TB *in vivo* and immunotherapy with both rAdhGLs and rAdhGLi is a very promising adjunct for chemotherapy against TB, especially for MDR-TB and XDR-TB.

After entering the lung, *M. tuberculosis* is engulfed immediately by alveolar macrophages and dendritic cells 11. Adaptive immune responses against TB are delayed to establish at least three weeks later and result in granuloma formation in the lung 12. *M. tuberculosis* might parasitize in the central area of the granuloma and become dormant 12 in immunocompromised hosts, for instance, dormant *M. tuberculosis* reactivates and replicates, and thus resulting in active pulmonary TB, formulating the main source of adult TB. Because both actively growing and dormant *M. tuberculosis* strains have been hypothesized to coexist in active pulmonary TB patients, the lengthy therapy with several drugs targeting different status of *M. tuberculosis* strains *in vivo* is necessary for current TB treatment 13, 14. Three new anti-TB drugs, namely bedaquiline, delamanid, and pretomanid have been approved for the treatment of MDR-TB patients over the past several decades 15-17, and several new and repurposed medications have also been introduced to develop shorter treatment regimens 18-21. However, effective controlling TB still faces great obstacles. First, most drugs are mainly used for actively replicating bacterial populations, only RIF and PZA have activity for dormant *M. tuberculosis* 22. Second, the step to develop novel drugs cannot keep up with the requirements of controlling TB, because new developed regimens still might trigger an amplification of drug resistance 23, 24. Therefore, the development of therapeutic vaccines such as bacilli or host-directed therapeutic methods to assist TB chemotherapy, have attracted increasing attention 25. Aimed to enhance host adaptive immune responses, immunotherapy with mycobacterial components, DNA vaccination, cytokines, antisense nucleotides or siRNA have been evaluated in preclinical or clinical trials 26-31. In this study, a rAd5-based rAdhGLs was successfully constructed. rAdhGLs effectively secrets granulysin from infected macrophages, which has a dose-dependent direct killing effect on extracellular *M. tuberculosis*. We further demonstrated that immunotherapy with both rAdhGLs and rAdhGLi could significantly inhibit the growth of *M. tuberculosis* H37Rv in the lung and spleen of infected SCID mice and prolongs their survival than rAdhGLs or rAdhGLi alone. Remarkably, our results also demonstrated that rAdhGLi and rAdhGLs plus chemotherapy achieved relapse-free cure after 4 months of therapy, suggesting that supplementation of standard antibiotics regimen with rAdhGLi and rAdhGLs could shorten the treatment duration needed to cure drug-susceptible TB patients. The treatment success attributes to the combined bactericidal action of rAdhGLi and rAdhGLs on intracellular and extracellular bacterial population. However, our study has its limitations. rAds were constructed based on the replication deficient (E1-deleted) adenovirus vectors and the expression of adenovirus-mediated transgene is transient. Several studies reported that the expression levels of the transgene in the lung peaked within a week after respiratory immunization, then decreased thereafter before being barely detectable two weeks later 32-34. This might be an explanation that immunotherapeutic regimens with rAds displayed a stronger ability to reduce bacterial load *in vivo* during the early stage of the treatment. In addition, distribution and location of the transgene expression in the lung and the dose of used rAd vector for treatment also influenced the effect of immunotherapy 9. The expression of granulysin in the lung distributed around the respiratory bronchiole and in the alveoli through intranasal instillation of rAdhGLi 9. Others also reported that transgene-expressed proteins were mainly localized to bronchial and alveolar epithelial cells, and to a lesser degree, alveolar macrophages in animal models 34-37. These findings indicate that rAds might result in incomplete clearance of *M. tuberculosis* in the infected lungs, as evidenced by the fact that treatment with different rAds alone could not eliminate *M. tuberculosis in vivo* in the current study. Under the circumstances, it is highly impracticable and almost impossible to replace current chemotherapeutic regimens with rAds-based immunotherapy alone for TB treatment.

Currently, the WHO new guidelines give the option of using longer or shorter regimens for MDR-TB treatment 17. The shorter MDR-TB regimen contains an injectable agent which is given for at least 4 months and may be less burdensome for patients, however, patients still have to endure months of painful injections and adverse events. The longer MDR-TB regimens have been designed and issued by WHO for several years and have been implemented in many countries worldwide. An all-oral long-term regimen is the preferred option and the injectable agents, kanamycin and capreomycin, are no longer recommended 17. Whereas, it has to be administered for a lengthy duration of at least 18-20 months, such that most patients are likely to give up the entire course of medication without close supervision 2. In this study, both rAdhGLs and rAdhGLi not only have a bactericidal effect on MDR-TB strains *ex vivo*, but also could lower organs bacterial load in MDR-TB infected mice. Moreover, only one dose of rAdhGLi and rAdhGLs combined with second-line drugs did actually reduce bacterial load in the lung and lung lesion after 1 month of treatment. Therefore, the combination treatment with adenovirus-mediated granulysin delivery is very promising to become a novel adjunctive treatment strategy for MDR-TB and XDR-TB.

## Supplementary Material

Supplementary figures and tables.

## Abbreviations

TB: Tuberculosis
MDR-TB: Multidrug-resistant TB
XDR-TB: Extensively drug-resistant TB
rAd5: recombinant adenovirus type 5
MOI: Multiplicity of infection
qRT-PCR: quantitative real-time PCR
i.n.: intranasally
s.c.: subcutaneously
ADC: Albumin-dextrose-catalase
CFU: Colony-forming unit
PFU: Plaque forming unit
tPA: tissue plasminogen activator
rAdhGLi: rAd5 expressing intracellular granulysin
rAdhGLs: rAd5 secreting extracellular granulysin
AdNull: Wild-type adenovirus serotype 5
rAds: rAdhGLi and rAdhGLs
RFP: Rifampin
INH: Isoniazid
PZA: Pyrazinamide
CFZ: Clofazimine
LFX: Levofloxacin
PAS: *p*-aminosalicylic acid
Kan: Kanamycin
2RHZ/4RH: 2-month of RFP+INH+PZA and 4-month of RFP+INH
RHZ: RFP+INH+PZA
CLPPK: CFZ+LFX+PZA+PAS+Kan
MDD: mean days to death
HE: hematoxylin-eosin
AFS: acid-fast staining
