# Supplementary material for: Enhanced tuberculosis clearance through the combination treatment with recombinant adenovirus-mediated granulysin delivery
Source: Theranostics. 2020 Aug 8;10(22):10046–56. doi: 10.7150/thno.48052 (PMC7481412; doi:10.7150/thno.48052)
Supplement: Supplementary file 1 — Supplementary figures and tables. [file thnov10p10046s1.pdf]

**Supplementary Materials**

**Table S1: Granulysin mRNA in rAds treated HEK293 cells was detected by qPCR.**

| Gene | Primer sequences (5'- 3') | Cycle parameters |
|---|---|---|
| GNLY | F: GATAAGCCCACCCAGAGAAG | 95 °C 30 sec; 95 °C 5 sec, 60 °C 34 sec, 40 cycles; 95 °C 1 min; 55 °C 1 min; 55-95 °C 4 sec, increase set point temperature by 0.5 °C |
| | R: GATCTGCTGGGCAGTTTCTC | |
| β-actin | F: TTCTACAATGAGCTGCGTG | 95 °C 30 sec; 95 °C 5 sec, 60 °C 34 sec, 40 cycles; 95 °C 1 min; 55 °C 1 min; 55-95 °C 4 sec, increase set point temperature by 0.5 °C |
| | R: CTCAAACATGATCTGGGTC | |

**Table S2: Therapeutic regimens of rAdhGLi and rAdhGLs on TB mice.**

| Groups | Number of mice | | | | | |
|---|---|---|---|---|---|---|
| | 2 M | 4 M | 6 M | 2+3 M† | 4+3 M | 6+3 M |
| PBS | 10 | 10 | 10 | | | |
| AdNull | 10 | 10 | 10 | | | |
| 2RHZ/4RH* | 10 | 10 | 10 | 10 | 10 | 10 |
| rAdhGLi | 10 | 10 | 10 | | | |
| rAdhGLs | 10 | 10 | 10 | | | |
| rAds | 10 | 10 | 10 | | | |
| rAdhGLi+2RHZ/4RH | 10 | 10 | 10 | 10 | 10 | 10 |
| rAdhGLs+2RHZ/4RH | 10 | 10 | 10 | 10 | 10 | 10 |
| rAds+2RHZ/4RH | 10 | 10 | 10 | 10 | 10 | 10 |

* 2RHZ/4RH means that mice were treat with 2-month of RFP+INH+PZA and 4-month of RFP+INH;

† 2+3 M, 4+3 M and 6+3 M mean that mice were killed 3 months after the end of each treatment.

**Table S3: Therapeutic regimens of rAdhGLi and rAdhGLs on MDR-TB mice.**

| Groups | Number of mice | | |
|---|---|---|---|
| | 15 D | 30 D | 60 D |
| PBS | 10 | 10 | |
| AdNull | 10 | 10 | |
| RHZ* | 10 | 10 | |
| rAdhGLi | 10 | 10 | |
| rAdhGLs | 10 | 10 | |
| rAds | 10 | 10 | |
| CLPPK† | 10 | 10 | 10 |
| rAdhGLi+CLPPK | 10 | 10 | |
| rAdhGLs+CLPPK | 10 | 10 | |
| rAds+CLPPK | 10 | 10 | 10 |

* RHZ means that mice were treat with RFP+INH+PZA; † CLPPK means that mice were treat with CFZ+LFX+PZA+PAS+Kan.

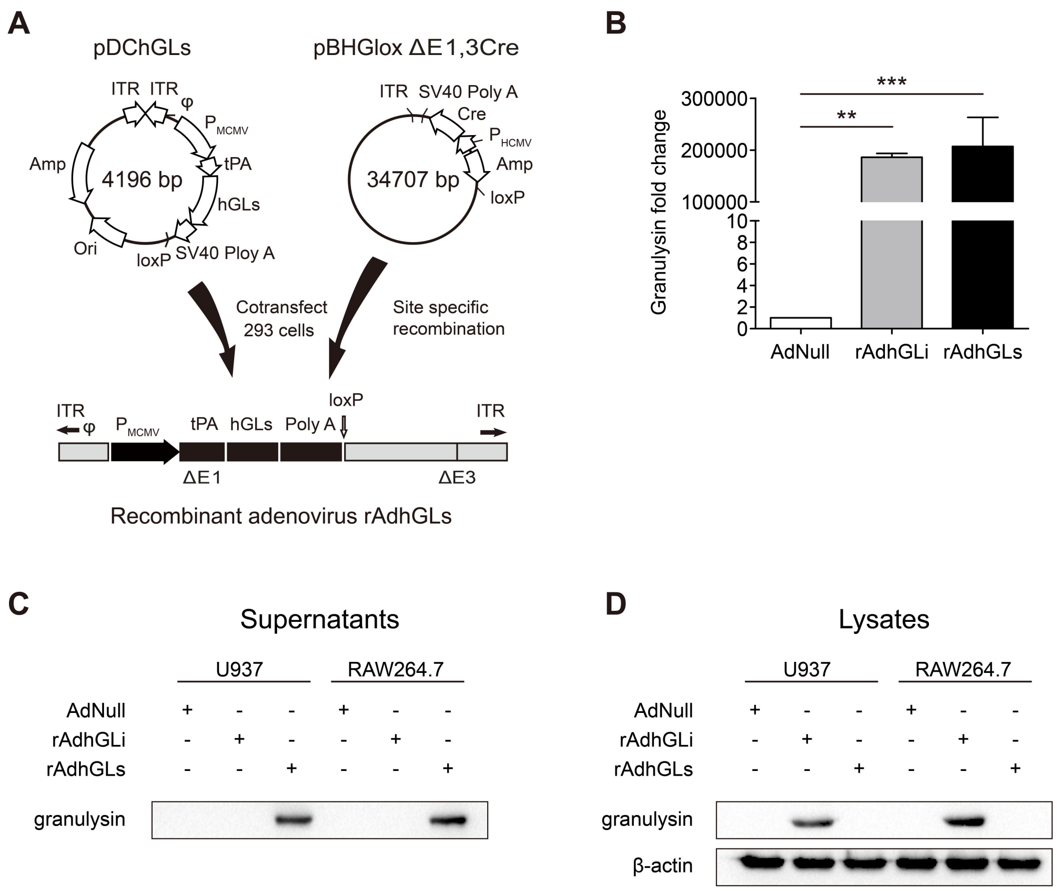


**Figure S1: Construction and identification of rAdhGLs. (A)** The construction procedure and the genome DNA structure of rAdhGLs. **(B)** The levels of granulysin mRNA in rAdhGLs-treated cells. rAdhGLi and AdNull were used as controls. The results were displayed as the Mean ± SD. Statistical significance was calculated by One-way ANOVA with Tukey's multiple comparison test. ** indicated $p$<0.01, and *** indicated $p$<0.001. **(C-D)** 72 h after infection, the expression of granulysin in the supernatants **(C)** or lysates **(D)** of rAdhGLs-treated U937 or RAW264.7 cells with different MOIs was confirmed by western blotting with mouse anti-granulysin antibody. rAdhGLi and AdNull were used as controls.

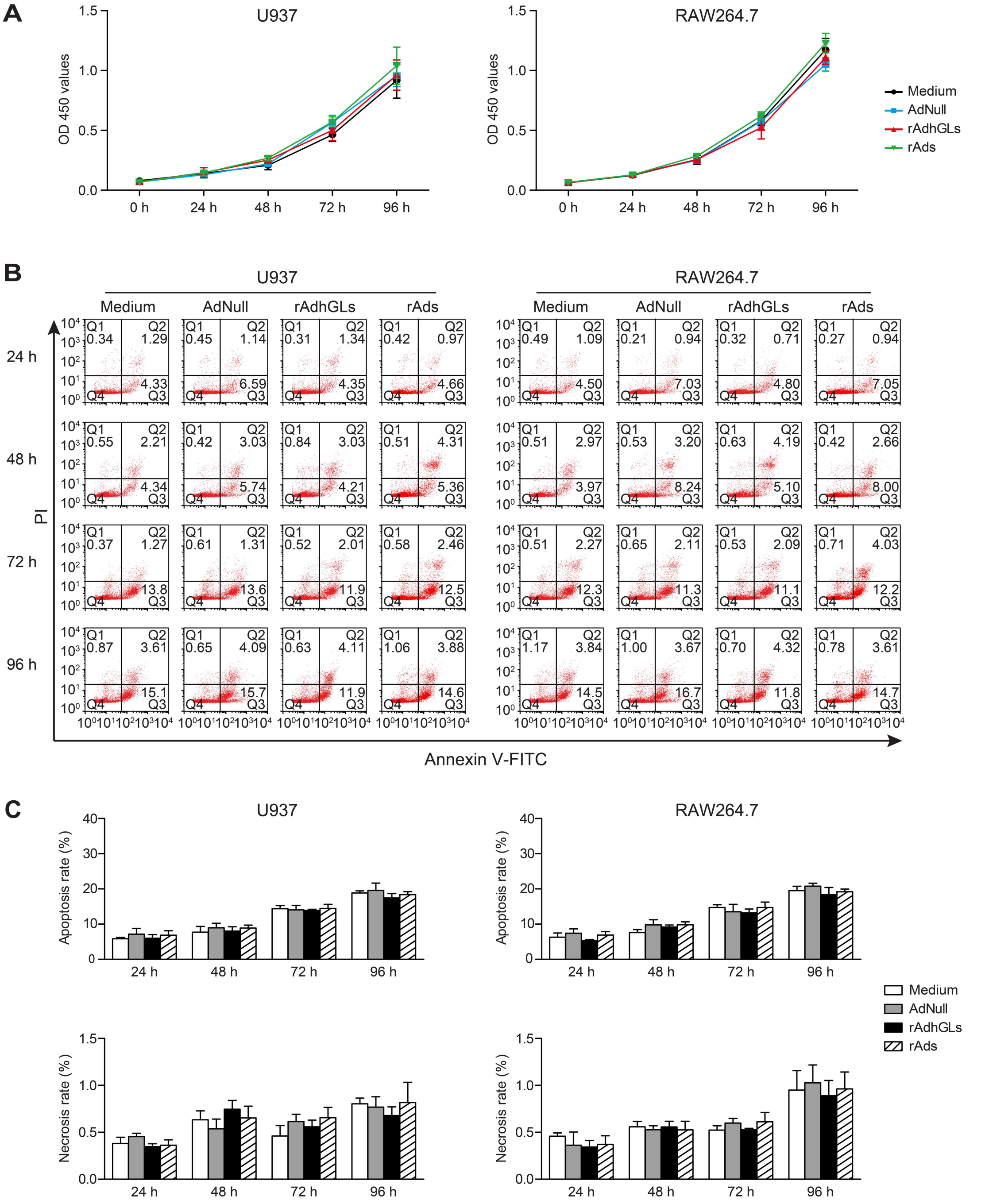


**Figure S2: The effects of rAdhGLs with or without rAdhGLi on the proliferation and death of macrophages. (A)** Cell proliferation was determined in U937 and RAW264.7 cells treated by rAdhGLs alone or combined with rAdhGLi (rAds) at indicated times. **(B)** The apoptotic and necrotic U937 and RAW264.7 cells were detected by Annexin V/PI assay after rAdhGLs or rAds treatment at indicated times. Culture medium and AdNull were used as negative controls. **(C)** Cell apoptotic and

necrotic rates. The results were displayed as the Mean ± SD of three repeats.
